# Prevalence of G6PD deficiency and G6PD variants amongst the southern Thai population

**DOI:** 10.7717/peerj.14208

**Published:** 2022-10-10

**Authors:** Manit Nuinoon, Rungnapha Krithong, Suputcha Pramtong, Piyawit Sasuk, Chompunuch Ngeaiad, Sathanan Chaimusik, Jiraporn Kanboonma, Orawan Sarakul

**Affiliations:** 1 Department of Medical Technology, School of Allied Health Sciences, Walailak University, Thasala, Nakhon Si Thammarat, Thailand; 2 Hematology and Transfusion Science Research Center (HTSRC), Walailak University, Thasala, Nakhon Si Thammarat, Thailand

**Keywords:** G6PD deficiency, Hemolytic anemia, Neonatal hyperbilirubinemia, G6PD variants, Southern Thai population

## Abstract

**Background:**

Glucose-6-phosphate dehydrogenase (G6PD) is an enzyme essential for NADPH production and protecting cells, especially red blood cells, from free radicals. The oxidative stress from drugs, chemicals, and infections can induce red blood cell hemolysis in G6PD deficiency patients, causing a genetic disorder.

**Objectives:**

This study aims to provide more information on G6PD deficiency prevalence and the G6PD variants in the southern Thai population.

**Methods:**

Five hundred and twenty healthy subjects in 14 provinces in the southern part of Thailand participated in the study. EDTA-blood samples were collected for a hematological parameters study, G6PD deficiency screening, and a molecular study for G6PD mutation. G6PD deficiency screening was tested using a fluorescent spot test. The types of G6PD mutation were identified by the allele-specific PCR method.

**Results:**

The prevalence of G6PD deficiency in southern Thailand was 6.1% (14/228) in males and 9.6% (28/292) in females. Two homozygous and 26 heterozygous G6PD deficiencies were found in females. G6PD Viangchan (871G>A) was the most common variant with 43%, followed by G6PD Mahidol (487G>A), 24% with an allele frequency of 0.025 and 0.012, respectively. Uncharacterized mutations existed in three samples. The study volunteers had anemia in 36.6% (107/292) females and 7.5% (17/228) males. Among G6PD deficiency subjects, only ten partial G6PD deficiency females had mild anemia.

**Conclusions:**

This study suggests that the prevalence of G6PD deficiency in southern Thailand aligns with that of other parts of Thailand. Newborn screening for G6PD deficiency is recommended for personal information and medical reference to prevent acute hemolysis from oxidative stressors.

## Introduction

Glucose-6-phosphate dehydrogenase (G6PD) is an essential enzyme in the pentose phosphate pathway, a step of the nicotinamide adenine dinucleotide phosphate (NADPH) generation (Adu-Gyasi et al., 2015). NADPH reduces glutathione, critical for red blood cell (RBC) protection from oxidative stress (Cappellini & Fiorelli, 2008). G6PD deficiency is an X-linked recessive inherited disorder, and symptomatic patients are mostly hemizygous in males whereas predominantly heterozygous and less common homozygous in females (Chen et al., 2018). G6PD deficiency is the most common enzyme deficiency, affecting at least 400 million individuals worldwide (Suryantoro, 2003). Point mutations of the G6PD encoded gene is located on chromosome X. Approximately 140 mutations (Hue et al., 2009) cause G6PD deficiencies, resulting in an amino acid substitution. The mutations lead to protein variants with different enzyme activity levels associated with biochemical and clinical phenotypes (Eggleston & Krebs, 1974). Especially in G6PD-deficient newborns, hyperbilirubinemia from red blood cell lysis may cause brain damage (Wang et al., 2010). However, G6PD deficiency screening is not conducted in all newborns in Thailand. The G6PD deficiency in a person or heterozygous female, which no evidence of hyperbilirubinemia or hemolysis, has inadequate information on protecting themselves from inducing substances. The distributions of G6PD deficiency differ globally, with a high prevalence in malaria-endemic areas (Phompradit et al., 2011). Primaquine is required for malaria treatment, especially for the radical cure of *Plasmodium vivax*, and *Plasmodium ovalae*, and reduces the transmission of *Plasmodium malariae* infection. The G6PD deficiency in persons with a malarial infection cannot be treated with a certain amount of primaquine because it may cause hemolytic anemia (Recht, Ashley & White, 2018, World Health Organization, 2015). The study of prevalence and G6PD variant in all parts of Thailand benefits the planning of malarial prevention and the safety of Primaquine treatment among these persons. Moreover, as G6PD deficiency is a genetic disorder, genetic consultation for couples may include providing more understanding and awareness.

In Thailand, the prevalence of G6PD deficiency is in the range of 3–18% (Tanphaichitr, 1999). The most common mutations in G6PD-deficient Thais are G6PD Viangchan (871G>A) and G6PD Mahidol (487G>A), respectively (Charoenkwan et al., 2014). The prevalence of G6PD deficiency in the south of Thailand was 3.33% in Trang province (Pattaranggoon & Yimtiang, 2014). The G6PD Viangchan is the most common type found in G6PD patients in Suratthani and Songkhla provinces (Laosombat et al., 2005, Jitueakul et al., 2018). However, according to previous reports, the prevalence of G6PD deficiency and its variants was not explored in all southern Thai provinces. This study aims to determine the prevalence of G6PD deficiency and the types of G6PD mutation in the southern Thai population. The findings from this study should support the previous data and provide more information on G6PD deficiency distribution in Thailand.

## Materials and Methods

### Subjects and blood collection

**Figure 1:**
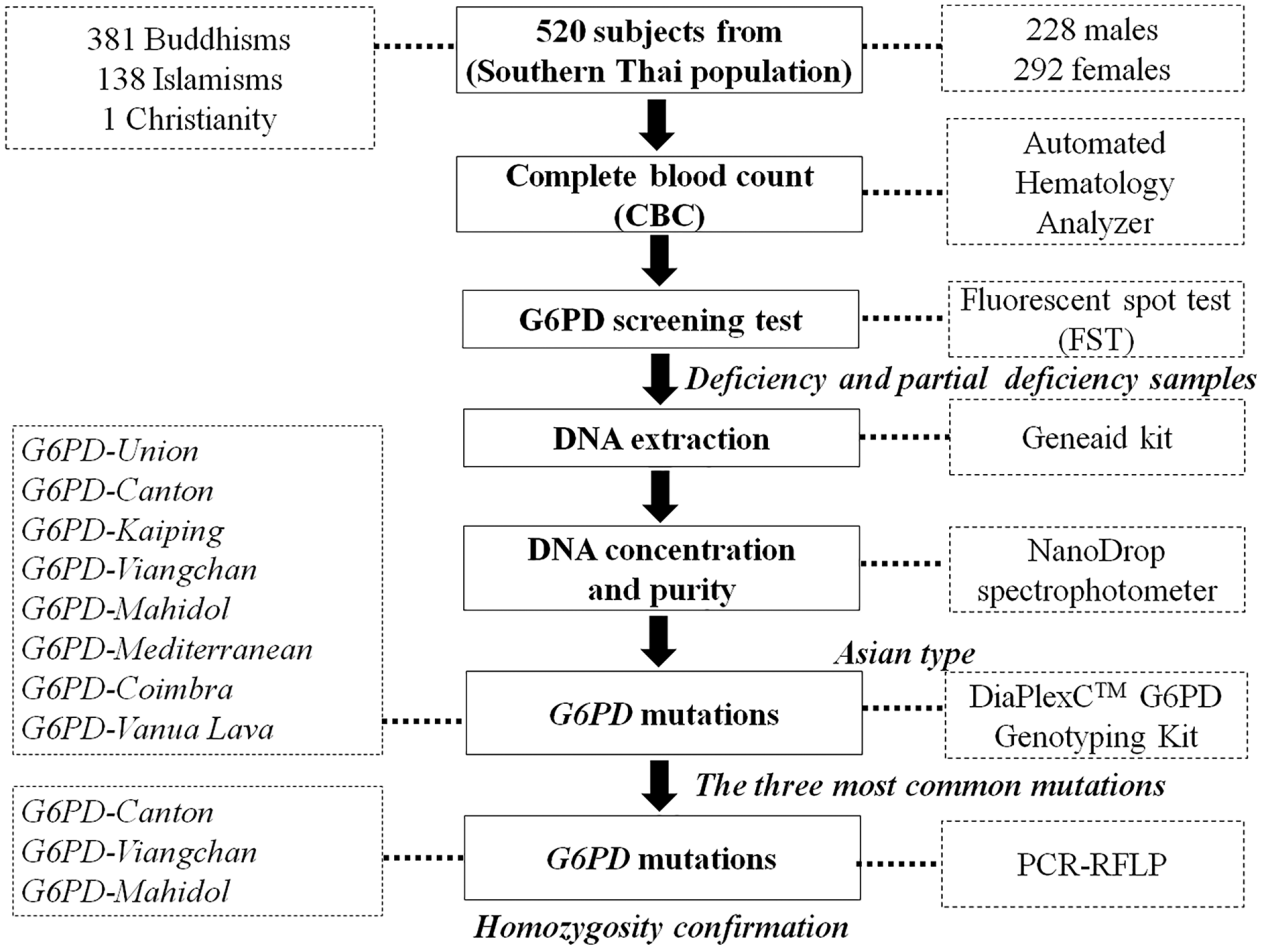
Sample workflow for laboratory testing. G6PD, glucose-6-phosphate dehydrogenase; PCR, polymerase chain reaction; RFLP, restriction fragment length polymorphism.

A cross-sectional study was conducted on 520 healthy volunteers (male:female = 228:292) in 14 provinces in the south of Thailand between July 2016 and August 2019. The interested persons were announced and informed of the recruitment criteria stating that the volunteer and their parents would be domiciled in southern Thailand. The number of volunteers from each province was calculated depending on the population in that province and the ratio of the total volunteers. Five hundred twenty volunteers were selected randomly. They were briefly informed and filled in their information in the questionnaire. They signed a consent form before the following processes. The 2.5 mL peripheral blood was drawn from each person and collected in ethylenediaminetetraacetic acid (EDTA) collection tubes without hemolysis. The blood sample of each subject was divided into 1.5 mL for G6PD deficiency screening and hematological parameters testing and 1.0 mL for DNA extraction for gene mutation analysis. The Human Research Committee of the Ethics Research Council, Walailak University (16/031 and WUEC-16-055-01) approved this research and informed consent. Figure 1 depicts the sample workflow of this study.

### Hematological parameters study

Hematological parameters were tested using Sysmex XN-1000 automated hematology analyzer (Sysmex Corporation, Kobe, Japan). The parameters included RBC count (RBC), hemoglobin (HBG), hematocrit (HCT), mean corpuscular volume (MCV), mean corpuscular hemoglobin (MCH), mean corpuscular hemoglobin concentration (MCHC), and red blood cell distribution width (RDW).

### G6PD screening test by Fluorescent spot test (FST)

G6PD deficiency screening was tested using a Fluorescent spot test (FST) (R&D Diagnostics Ltd., Papagos, Greece). The protocol was clearly described in the previous study (Krithong et al., 2020). The G6PD variants further identified the G6PD deficiency and partial deficiency.

### Detection of G6PD gene mutation

Genomic DNA was extracted from peripheral blood leukocytes using a Geneaid kit (Geneaid Biotech Ltd, New Taipei City, Taiwan). The DNA concentration of each sample was quantified with the NanoDrop spectrophotometer (Thermo Scientific, Waltham, MA, USA). The G6PD mutations in G6PD-deficient and partially deficient samples were identified using multiplex allele-specific PCR, DiaPlexC™ G6PD Genotyping Kit (Asian type) (SolGent Co., LTD., Daejeon, Korea). The kit allows screening of eight mutations of Asian types, including Union (1360C>T), Canton (1376G>T), Kaiping (1388G>A), Viangchan (871G>A), Mahidol (487G>A), Mediterranean (563C>T), Coimbra (592C>T) and Vanua Lava (383T>C). The PCR reaction comprised 12.5 μL of 2X Multiplex PCR Smart mix (G6PD-Asian type), 2 μL of primer mixture (G6PD-Asian type), nuclease-free water, and DNA template (25–50 ng). PCR thermocycling conditions were as follows: initial denaturation at 95 °C for 15 min; followed by 30 cycles of 30 s at 95 °C, 30 s at 60 °C, and 40 s at 72 °C; followed by a final extension at 72 °C for 5 min. PCR products of each sample and DNA marker were analyzed by 3% agarose gel electrophoresis for the variants. After staining the gel with ethidium bromide for 5 min, the sizes of PCR products were detected under UV light with an ultraviolet transilluminator. Finally, PCR-RFLP was used for secondary confirmation of heterozygous female, hemizygous male, and homozygous female in the first three common G6PD variants (G6PD Viangchan, G6PD Mahidol, and G6PD Canton) (Nuchprayoon, Sanpavat & Nuchprayoon, 2002).

### Statistical analysis

Data were analyzed using SPSS v.17 software. Hematological parameters were calculated for the mean, SD, median, and prevalence. The Kolmogorov-Smirnov test was used to test the normality of the data. The statistical difference in hematological parameters between the groups was calculated using the T-Test Independent statistics for normal distribution and the Mann-Whitney U test for non-normal distribution. The 100% stacked column was conducted using Infogram (https://infogram.com/).

## Results

### Demographic data and G6PD screening test

The data were collected from 520 subjects in 14 provinces of Southern Thailand, including 228 males and 292 females. The age of the subjects was in the range of 20–25 years old, with religious backgrounds of 73.3% being Buddhist and 26.5% being Muslim (Table 1). FST tested the G6PD deficiency. The prevalence of G6PD deficiency was 6.1% (14/228) for males. For females, the G6PD deficiency and partial or intermediate G6PD deficiency was detected in two and 26 persons, respectively. Thus, the prevalence in females was 9.6% (28/292). No significant differences (*p* > 0.05) existed in hematological parameters of G6PD deficiency, G6PD partial, and normal G6PD (Table 2). Mild anemia was found in 10 persons with partial G6PD deficiency in females but not in G6PD deficient males. Additionally, the prevalence of G6PD deficiency was 9.4% (13/138) in Muslims and 7.6% (29/381) in Buddhists.

**Table 1:** Geographic characteristics of participants from 14 provinces in the south of Thailand.

Provinces, *n* (%)	Participants		Religion			G6PD status	
	Male, *n* (%)	Female, *n* (%)	Buddhism, *n* (%)	Islamism, *n* (%)	Christianity, *n* (%)	Partial G6PD deficiency, *n* (%)	G6PD deficiency, *n* (%)
Nakhon Si Thammarat, 145 (27.9)	65 (12.5)	80 (15.4)	127 (24.4)	19 (3.7)	–	6 (1.2)	4 (0.8)
Trang, 40 (7.7)	20 (3.8)	20 (3.8)	30 (5.8)	9 (1.7)	1 (0.2)	4 (0.8)	1 (0.2)
Songkhla, 53 (10.2)	26 (5.0)	27 (5.2)	32 (6.2)	20 (3.8)	–	3 (0.6)	3 (0.6)
Suratthani, 50 (9.6)	24 (4.6)	26 (5.0)	41 (7.9)	9 (1.7)	–	3 (0.6)	1 (0.2)
Krabi, 33 (6.4)	12 (2.3)	21 (4.0)	21 (4.0)	12 (2.3)	–	2 (0.4)	0 (0.0)
Phatthalung, 28 (5.4)	11 (2.1)	17 (3.3)	20 (3.8)	8 (1.5)	–	1 (0.2)	1 (0.2)
Chumphon, 24 (4.6)	9 (1.7)	15 (2.9)	22 (4.2)	2 (0.4)	–	1 (0.2)	0 (0.0)
Narathiwat, 37 (7.1)	15 (2.9)	22 (4.2)	15 (2.9)	22 (4.2)	–	2 (0.4)	0 (0.0)
Satun, 22 (4.2)	10 (1.9)	12 (2.3)	14 (2.7)	8 (1.5)	–	1 (0.2)	1 (0.2)
Pattani, 39 (7.5)	14 (2.7)	25 (4.8)	21 (4.0)	18 (3.5)	–	1 (0.2)	3 (0.6)
Ranong, 13 (2.5)	5 (1.0)	8 (1.5)	12 (2.3)	1 (0.2)	–	0 (0.0)	1 (0.2)
Phangnga, 13 (2.5)	5 (1.0)	8 (1.5)	11 (2.1)	2 (0.4)	–	0 (0.0)	1 (0.2)
Yala, 15 (2.9)	8 (1.5)	7 (1.3)	8 (1.5)	7 (1.3)	–	1 (0.2)	0 (0.0)
Phuket, 8 (1.5)	4 (0.8)	4 (0.8)	7 (1.3)	1 (0.2)	–	1 (0.2)	0 (0.0)
Total, 520 (100.0)	228 (43.8)	292 (56.2)	381 (73.3)	138 (26.5)	1 (0.2)	26 (5.0)	16 (3.1)

**Notes:**

The data indicates the general data of all participants including province, gender, religions and G6PD status.

Values are presented as *n* (%). G6PD, Glucose-6-phosphate dehydrogenase.

**Table 2:** Prevalence of G6PD deficiency and hematological parameters in the study population.

Gender/G6PD status (*n*)	Prevalence (%)	RBC (10^6^/µL)	HGB (g/dL)	HCT (%)	MCV (fL)	MCH (pg)	MCHC (g/dL)	RDW (%)
Male (228)								
– Normal (214)	93.9	5.28 ± 0.54	14.5 ± 1.23	42.9 ± 3.40	81.7 ± 5.85	27.6 ± 2.32	33.8 ± 1.26	13.5 ± 1.66
– Deficiency (14)	6.1	5.09 ± 0.44	14.7 ± 1.08	43.0 ± 3.14	83.8 ± 4.29	27.8 ± 3.41	34.2 ± 0.84	12.9 ± 1.26
*P*-value^a^		0.116	0.613	0.867	0.271	0.485	0.196	0.116
Female (292)								
– Normal (264)	90.4	4.65 ± 0.45	12.1 ± 1.18	36.8 ± 3.15	79.6 ± 7.80	26.3 ± 3.03	32.9 ± 1.26	14.1 ± 1.93
– Partial deficiency (26)	8.9	4.60 ± 0.47	12.1 ± 1.33	36.6 ± 3.02	79.9 ± 7.03	26.4 ± 3.05	32.9 ± 1.66	13.8 ± 1.87
– Deficiency (2)	0.7	4.82, 4.02	13.2, 12.0	39.5, 36.6	82.0, 86.1	27.4, 28.9	33.4, 33.5	13.5, 14.7
*P*-value^b^		0.598	0.510	0.526	0.998	0.996	0.675	0.527

**Notes:**

The participants were grouped by sex and G6PD deficiency status. The data of the hematological parameters of each group were analyzed and were presented as mean ± SD. The different of each parameter between normal and G6PD deficiency was statistical analyzed.

Values are presented as *n*, %, mean ± SD, or raw data where appropriate. RBC, red blood cell; HGB, hemoglobin; HCT, hematocrit; MCV, mean corpuscular volume; MCH, mean corpuscular hemoglobin; MCHC, mean corpuscular hemoglobin concentration; RDW, red cell distribution width; fL, femtoliter; g/dL, gram per deciliter.

a Statistical comparison was done using the Mann-Whitney U test and *P*-values between normal *vs* deficiency groups of males.

b Statistical comparison was done using the Mann-Whitney U test and *P*-values between normal *vs* partial deficiency groups of females.

### Characterization of G6PD mutations

**Figure 2:**
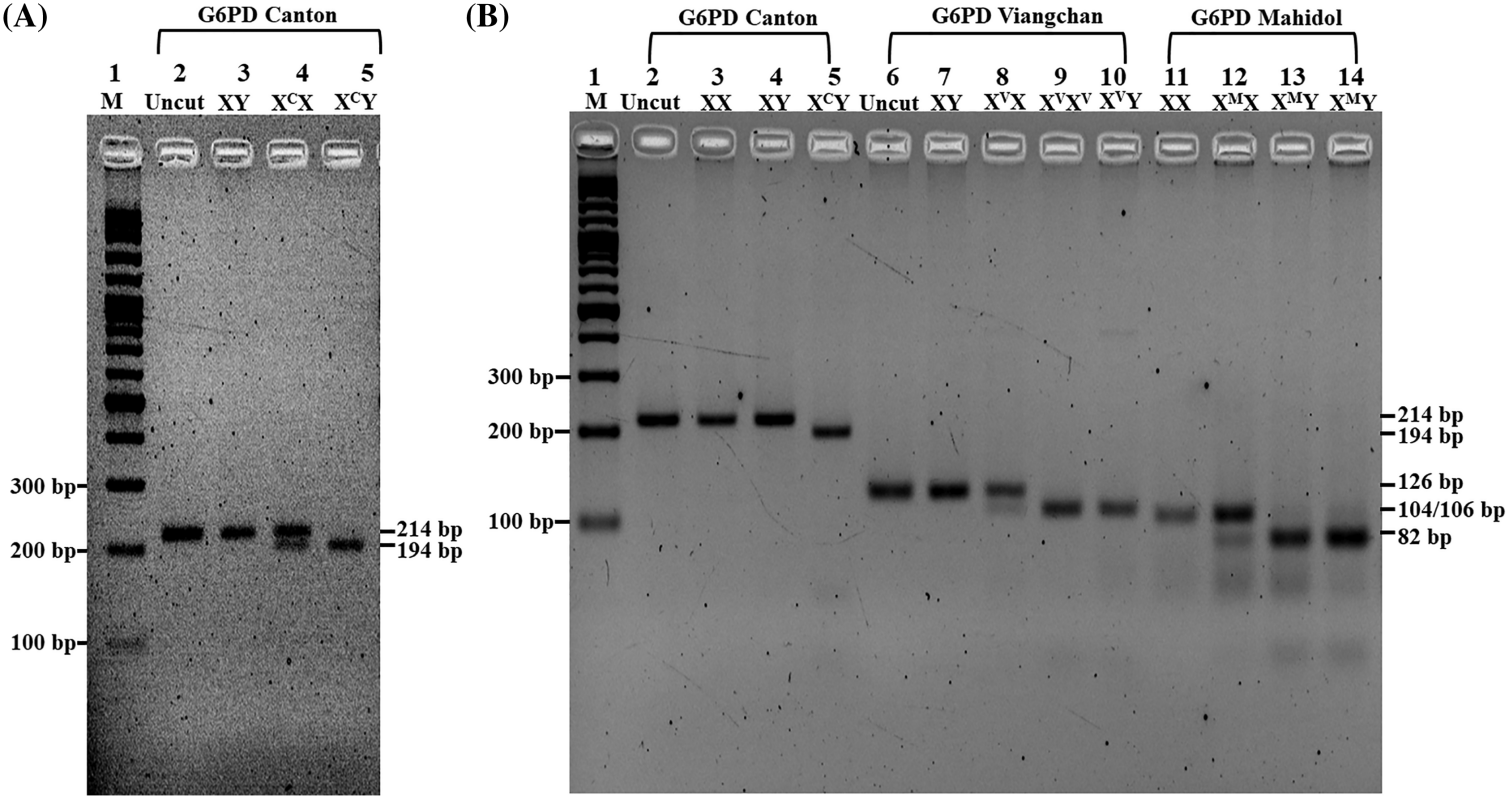
Characterization of G6PD mutations by PCR-RFLP. Two percent Agarose gel electrophoresis of PCR-RFLP from G6PD Canton (X^C^), G6PD Viangchan (X^V^), and G6PD Mahidol (X^M^). (A) Lane 1, standard marker (100 bp DNA ladder); lane 2, uncut normal control (XY); lane 3, cut normal control (XY); lane 4, heterozygous G6PD Canton (X^C^X); lane 5, hemizygous G6PD Canton (X^C^Y). (B) Lane 1, standard marker (100 bp DNA ladder); lane 2, uncut normal control (XX); lane 3, cut normal control (XX); lane 4, cut normal control (XY); lane 5, hemizygous G6PD Canton (X^C^Y); lane 6, uncut normal control (XY), lane 7, cut normal control (XY); lane 8, heterozygous G6PD Viangchan (X^V^X); lane 9, homozygous G6PD Viangchan (X^V^X^V^), lane 10, hemizygous G6PD Viangchan (X^V^Y); lane 11, cut normal control (XX); lane 12, heterozygous G6PD Mahidol (X^M^X) lanes 13 and 14, hemizygous G6PD Mahidol (X^M^Y).

A total of 42 G6PD deficiency subjects (14 males and 28 females) were analyzed for eight types of G6PD gene mutation by DiaPlexC™ G6PD Genotyping Kit (Asian type) and further confirmation by PCR-RFLP in the first three common mutations. PCR-RFLP with *Afl*II restriction enzyme revealed good discrimination among three genotypes (XY, X^C^X, and X^C^Y) of the G6PD Canton (Fig. 2A) in addition to PCR-RFLP with *Xba*I and *Hind*III restriction enzymes for genotypic characterization of G6PD Viangchan and G6PD Mahidol, respectively (Fig. 2B). Figures S1 and S2 depict the product sizes of each variant in G6PD deficiency and partial deficiency.

### The frequency of G6PD variants in the southern Thai population

**Figure 3:**
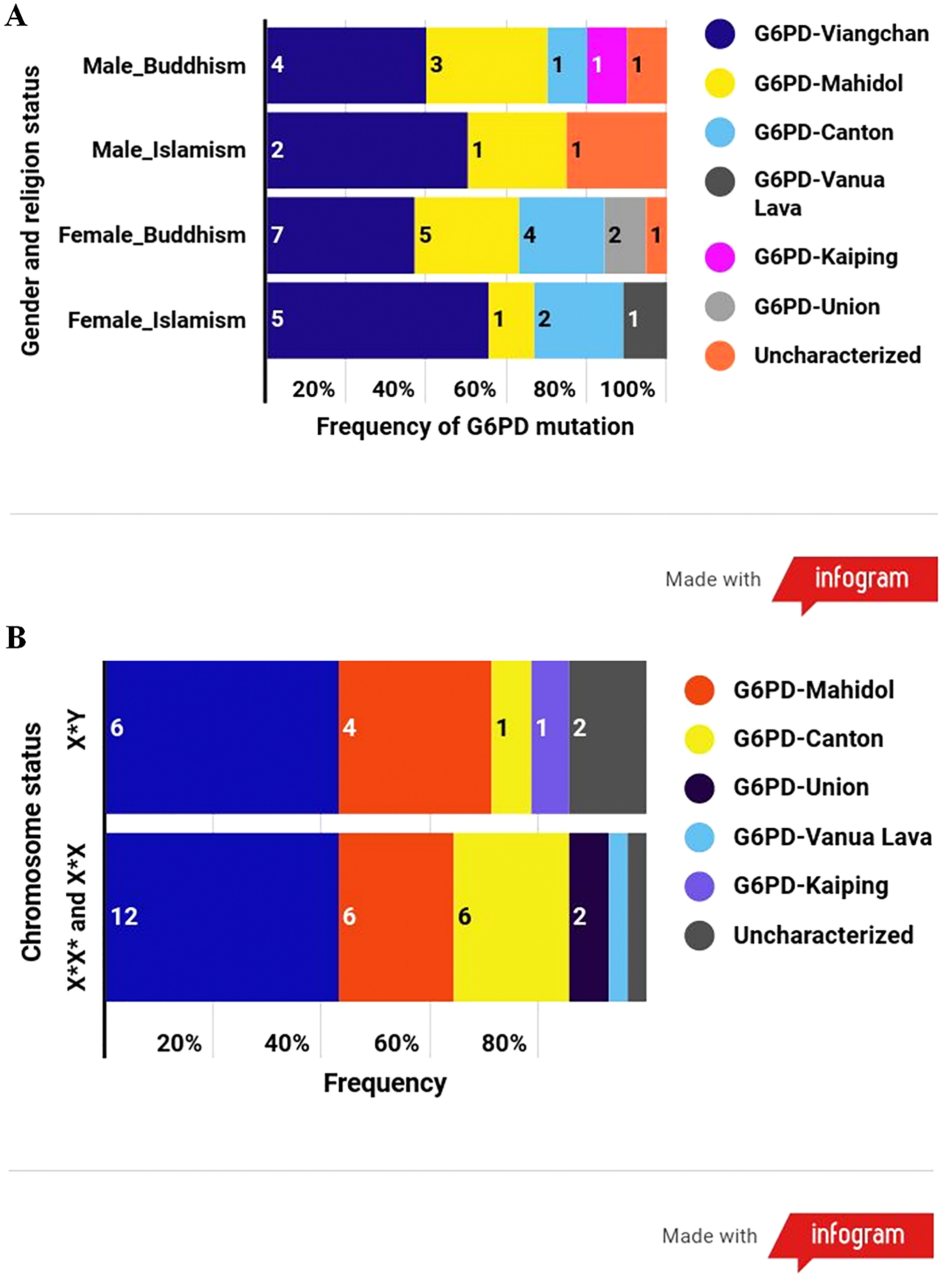
The frequency of G6PD variants according to different categories. (A) Frequency of G6PD mutations according to the combination of gender and religion. (B) Frequency of G6PD mutations according to the number of X chromosome (gender).

The results disclosed that the most common G6PD variant in this population was G6PD Viangchan (43%; allele frequency = 0.025), followed by G6PD Mahidol (24%; 0.012), G6PD Canton (17%; 0.009), G6PD Union (5%; 0.002), G6PD Vanua Lava (2%; 0.001), and G6PD Kaiping (2%; 0.001). Uncharacterized mutations existed in three samples (Table 3). Among G6PD-deficient samples, G6PD-Viangchan was the most common G6PD variant in every category according to the combination of gender and religion and followed by G6PD-Mahidol (Fig. 3A). G6PD mutational spectrum illustrated a similar pattern between males and females (Fig. 3B).

**Table 3:** G6PD variants and allele frequency in 42 G6PD-deficient samples.

G6PD variants	Male (228)		Female (292)				No. of X* Chr.	Total X or X* Chr.	Allele frequency
	Hemizygote (14)	Normal (214)	Homozygote (2)	Heterozygote (26)		Normal (264)			
	(X*Y)	(XY)	(X*X*)	X*	X	(XX)			
G6PD-Viangchan	6	0	4	10	0	0	20	20	0.025
G6PD-Mahidol	4	0	0	6	0	0	10	10	0.012
G6PD-Canton	1	0	0	6	0	0	7	7	0.009
G6PD-Union	0	0	0	2	0	0	2	2	0.002
G6PD-Vanua Lava	0	0	0	1	0	0	1	1	0.001
G6PD-Kaiping	1	0	0	0	0	0	1	1	0.001
G6PD-Uncharacterized	2	0	0	1	0	0	3	3	0.004
G6PD-Normal	0	214	0	0	26	528	0	768	0.946
Total	14	214	4	26	26	528	44	812	1.000

**Notes:**

G6PD variants of all G6PD deficiency samples were calculated for allele frequency.

G6PD, Glucose-6-phosphate dehydrogenase.

X* represented the *G6PD* gene mutation.

### Red blood cell parameters in participants with anemia and different G6PD status

Table 4 demonstrates the mean ± SD of 228 males and 292 females of hematological parameters. Subjects were grouped into healthy (76.2%, 396/520) and anemia (23.8%, 124/520) by hemoglobin value; anemia has a hemoglobin value of less than 12 g/dL in females and less than 13 g/dL in males. Anemia was found in 36.6% (107/292) females and 7.5% (17/228) males. Interestingly, this study found two cases with homozygous G6PD-Viangchan (X^V^X^V^). In the first case, a 19-year-old from Ranong province, her complete blood count was as follows: red cell count 4.82 × 10^12^/L, Hb level 13.2 g/dL, hematocrit 39.5%, MCV 82.0 fL, MCH 27.4 pg, MCHC 33.4 g/dL, and RDW 13.5%. The second case was a 20-year-old from Songkhla province; her complete blood count was as follows: red cell count 4.02 × 10^12^/L, Hb level 12.0 g/dL, hematocrit 36.6%, MCV 86.1 fL, MCH 28.9 pg, MCHC 33.5 g/dL, and RDW 14.7 % as in Table 2.

**Table 4:** The hematological parameters of the normal group and anemia group categorized by gender.

Gender	Anemia/Normal	RBC^b^ (×10^6^/µL)	HGB^a^ (g/dL)	HCT^a^ (%)	MCV^a^ (fL)	MCH^a^ (pg)	MCHC^a^ (g/dL)	RDW^a^ (%)
Male	Anemia (*n* = 17)	4.86 ± 0.6	12.1 ± 0.8	37.8 ± 3.2	78.4 ± 7.5	25.6 ± 3.1	32.6 ± 1.6	14.8 ± 3.3
	Normal (*n* = 211)	5.30 ± 0.5	14.7 ± 1.0	43.3 ± 3.0	82.1 ± 5.6	27.8 ± 2.2	34.0 ± 1.1	13.4 ± 1.4
	*P*-value	0.005	0.000	0.000	0.072	0.005	0.000	0.030
Female	Anemia (*n* = 107)	4.58 ± 0.5	11.0 ± 0.9	34.0 ± 2.4	74.9 ± 8.9	24.3 ± 3.4	32.3 ± 1.5	15.2 ± 2.3
	Normal (*n* = 185)	4.68 ± 0.4	12.8 ± 0.7	38.5 ± 2.2	82.4 ± 5.3	27.4 ± 2.0	33.3 ± 1.0	13.3 ± 1.1
	*P*-value	0.007	0.000	0.000	0.000	0.000	0.000	0.000

**Notes:**

Participants were grouped by gender. The normal and anemia were grouped in each gender by hemoglobin level. Hematological parameters of normal and anemia groups were statistical analyzed.

a Mann-Whitney U Test.

b Independent Samples T-Test

A *P* value < 0.05 was considered significant.

## Discussion

G6PD deficiency is the most common enzyme deficiency worldwide (Bubp, Jen & Matuszewski, 2015). The prevalence of G6PD deficiency substantially varies across the world. In Thailand, the prevalence was 3–18%, with different percentages in each part of the country. Northeastern and northern Thailand have deficiencies of 22% and 17%, respectively (Charoenkwan et al., 2014; Kittiwatanasarn et al., 2003). The previous study of G6PD deficiency in neonatal for the Southern part showed a high prevalence of G6PD deficiency in male neonatal and hyperbilirubinemia in newborns mainly resulting from a G6PD deficiency (Pattaranggoon & Yimtiang, 2014; Tanphaichitr, 1999; Tuchinda et al., 1968). This study reported the prevalence of G6PD deficiency in healthy adults in the Southern area. The finding was consistent with other reports in which the prevalence was 6.1% and 9.6% in males and females, respectively. However, this report revealed a higher prevalence of G6PD deficiency in females than males because of the criteria set by the FST report. The World Health Organization (WHO) recommended the G6PD deficiency screening method of FST (Ley et al., 2017). This study used this test for G6PD deficiency screening and prevalence evaluation. The results of NADPH production included being fluorescent under the UV light, time to provide the NADPH synthesis in different G6PD levels, or activity in blood samples. These could divide the G6PD deficiency (in hemizygous male and homozygous female), and partial G6PD deficiency (heterozygous female) in healthy people’s blood samples (Thielemans et al., 2018). PCR-based methods confirmed all these deficiency samples.

The base substitution mainly causes gene mutation in G6PD deficiency. As the G6PD gene is 18 kb, the mutation in each point of the G6PD gene leads to a difference in severity (Gómez-Manzo et al., 2016). The class or severity of the G6PD deficiency is based on the activity of the remaining enzymes. For class I, the enzyme activity is less than 1% of the standard range and less than 10% in class II, whereas no abnormal signs were depicted in class V. Common G6PD mutations in Southeast Asia are G6PD Union (1360C>T), G6PD Canton (1376G>T), G6PD Kaiping (1388G>A), G6PD Viangchan (871G>A), G6PD Mahidol (487G>A), G6PD Gaohe (95 A>G), G6PD Chatham (1003 G>A), G6PD Coimbra (592C>T) and G6PD Vanua Lava (383T>C) (Iwai et al., 2001). In Thailand, many studies reported that G6PD Viangchan (871G>A) and G6PD Mahidol (487G>A) are the most commonly found (Laosombat et al., 2005; Nuchprayoon, Sanpavat & Nuchprayoon, 2002). This study also found that these two variants are the most common among the southern Thai population. The prevalence of G6PD deficiency in Thai Muslims and Buddhists in this study is 9.42% and 7.61%, respectively. The G6PD Viangchan is the most common type in both. The G6PD Viangchan was the most common type in Malaysian Malays (Ainoon et al., 2003; Yusoff et al., 2003). Most Thai Muslims have Malay ancestry (Kutanan et al., 2014), and the most common G6PD variant of Thai Muslims from this study was the same as in Malaysian Malays. Consistent with the Malaysian Chinese, the most common types are Canton and Kaiping. (Ainoon et al., 2004). Unidentified variants exist in three G6PD deficiency samples. Because the primers used in the multiplex allele-specific PCR in this study, cover only eight common mutations in Southeast Asia, the unidentified samples might be rare types in this area. Because the G6PD gene is located on chromosome X, the clinical signs are mainly present in hemizygous males and homozygous females when G6PD deficiency patients are exposed to oxidative inducing agents or infections (Hsieh et al., 2013). However, it mainly does not affect in heterozygous females (van den Broek, Heylen & van den Akker, 2016). This study’s limitation is the sample recruitment from certain provinces with small sample size. G6PD deficiency was classified by the level of residual enzyme activity. Thus, the classification will be revised based on the median residual enzyme activity (World Health Organization, 2022). G6PD Mahidol, the G6PD Canton, and the G6PD Viangchan were WHO Class III mutations, moderating deficiency, and were not so severe for primaquine treatment for *P. vivax* and *P. ovale* patients (Phompradit et al., 2011). However, the prevention from the oxidative agents should be informed to all. The prevalence of G6PD deficiency and G6PD variants has been reported in the southern Thai population. However, those reports recruited the samples from one province and five provinces with a high prevalence of malaria (Pattaranggoon & Yimtiang, 2014, Khammanee et al., 2022) with a retrospective study. The samples from all provinces located in southern Thailand were collected in a cross-sectional study in this study. However, the limitations of this study are still insufficient sample size in some provinces. It should be helpful in area-based research with differences in ethnicity. Therefore, this study could be confirmed with the evidence of the prevalence of G6PD deficiency in the southern Thai population, and G6PD-Viangchan and G6PD-Mahidol are the two most common G6PD mutations, consistent with previous reports. Our results provide valuable data for planning and informing people with G6PD deficiency, including their parents, to protect themselves from further exposure to hemolytic stimulants to achieve the well-being of G6PD deficiency persons.

## Conclusions

The data reveal a high prevalence of partial G6PD deficiency in heterozygous females. This heterozygous group is the carrier of the abnormal gene to their child. Thus, G6PD screening should be performed for all family members of G6PD-deficient patients to provide information for self-protection from oxidative stress.

## Supplemental Information

10.7717/peerj.14208/supp-1 Supplemental Information 1 Raw data and statistical data. Raw data of all participants including general data, G6PD deficiency screening results, hematological parameters.

10.7717/peerj.14208/supp-2 Supplemental Information 2 PCR product of G6PD variant in G6PD deficiency samples. The pattern of PCR products of G6PD deficiency cases was analyzed by 3% gel electrophoresis**. **Lane 1, standard marker; lane 2, mutant type control; lane 3, wild type control; lane 4, non-template control; lane 5, G6PD normal; lane 6, G6PD Canton (681 bp); lane 7 G6PD Kaiping (557 bp); lane 8 G6PD Viangchan (501 bp); lane 9 G6PD Mahidol (337 bp) and lane 10 no detectable mutation by PCR used in this study.

10.7717/peerj.14208/supp-3 Supplemental Information 3 The pattern of PCR products of G6PD partial deficiency cases analyzed by 3% gel electrophoresis. The pattern of PCR products of G6PD partial deficiency cases analyzed by 3% gel electrophoresis. Lane 1, standard marker; lane 2, mutant type control; lane 3, wild type control; lane 4, non-template control; lane 5, G6PD normal; lane 6, G6PD Vanua Lava (154 bp); lane 7, G6PD Viangchan (501 bp); lane 8, G6PD Mahidol (337 bp); lane 9, Canton (681 bp); lane 10, G6PD Union (803 bp) and lane 11, no detectable mutation by PCR used in this study.

## Additional Information and Declarations

### Data Availability

The following information was supplied regarding data availability: The raw measurements are available in the Supplemental Files.

## References

[ref-1] Adu-Gyasi D, Asante KP, Newton S, Dosoo D, Amoako S, Adjei G, Amoako N, Ankrah L, Tchum SK, Mahama E, Agyemang V, Kayan K, Owusu-Agyei S (2015). Evaluation of the diagnostic accuracy of CareStart G6PD deficiency rapid diagnostic test (RDT) in a malaria endemic area in Ghana, Africa. PLOS ONE.

[ref-2] Ainoon O, Boo NY, Yu YH, Cheong SK, Hamidah HN, Lim JH (2004). Complete molecular characterisation of glucose-6-phosphate dehydrogenase (G6PD) deficiency in a group of Malaysian Chinese neonates. Malaysian Journal of Pathology.

[ref-3] Ainoon O, Yu YH, Amir Muhriz AL, Boo NY, Cheong SK, Hamidah NH (2003). Glucose-6-phosphate dehydrogenase (G6PD) variants in Malaysian Malays. Human Mutation.

[ref-4] Bubp J, Jen M, Matuszewski K (2015). Caring for glucose-6-phosphate dehydrogenase (G6PD)-deficient patients: implications for pharmacy. P & T: a peer-reviewed journal for formulary management.

[ref-5] Cappellini MD, Fiorelli G (2008). Glucose-6-phosphate dehydrogenase deficiency. Lancet.

[ref-6] Charoenkwan P, Tantiprabha W, Sirichotiyakul S, Phusua A, Sanguansermsri T (2014). Prevalence and molecular characterization of glucose-6-phosphate dehydrogenase deficiency in northern Thailand. The Southeast Asian Journal of Tropical Medicine and Public Health.

[ref-7] Chen Y, Xiu W, Dong Y, Wang J, Zhao H, Su Y, Zhou J, Zeng Y, Li H, Wo J, Lin F, Zhang H, Chen H, Yang C, Zhu W (2018). Mutation of glucose-6-phosphate dehydrogenase deficiency in Chinese Han children in eastern Fujian. Medicine.

[ref-8] Eggleston LV, Krebs HA (1974). Regulation of the pentose phosphate cycle. Biochemical Journal.

[ref-9] Gómez-Manzo S, Marcial-Quino J, Vanoye-Carlo A, Serrano-Posada H, Ortega-Cuellar D, González-Valdez A, Castillo-Rodríguez RA, Hernández-Ochoa B, Sierra-Palacios E, Rodríguez-Bustamante E, Arreguin-Espinosa R (2016). Glucose-6-phosphate dehydrogenase: update and analysis of new mutations around the world. International Journal of Molecular Sciences.

[ref-10] Hsieh Y-T, Lin M-H, Ho H-Y, Chen L-C, Chen C-C, Shu J-C (2013). Glucose-6-phosphate dehydrogenase (G6PD)-deficient epithelial cells are less tolerant to infection by Staphylococcus aureus. PLOS ONE.

[ref-11] Hue NT, Charlieu JP, Chau TTH, Day N, Farrar JJ, Hien TT, Dunstan SJ (2009). Glucose-6-phosphate dehydrogenase (G6PD) mutations and haemoglobinuria syndrome in the Vietnamese population. Malaria Journal.

[ref-12] Iwai K, Hirono A, Matsuoka H, Kawamoto F, Horie T, Lin K, Tantular IS, Dachlan YP, Notopuro H, Hidayah NI, Salim AM, Fujii H, Miwa S, Ishii A (2001). Distribution of glucose-6-phosphate dehydrogenase mutations in Southeast Asia. Human Genetics.

[ref-13] Jitueakul S, Buncherd H, Thawornpan P, Win Tung A, Thanapongpichat S (2018). Characterization of G6PD genotypes in G6PD deficiency patients from Suratthani Hospital, Thailand. Journal of Associated Medical Sciences.

[ref-14] Khammanee T, Sawangjaroen N, Buncherd H, Tun AW, Thanapongpichat S (2022). Prevalence of glucose-6-phosphate dehydrogenase (G6PD) deficiency among malaria patients in Southern Thailand: 8 years retrospective study. The Korean Journal of Parasitology.

[ref-15] Kittiwatanasarn P, Louicharoen C, Sukkapan P, Nuchprayoon I (2003). Glucose-6-phosphate dehydrogenase deficiency in Northeastern Thailand: prevalence and relationship to neonatal jaundice. Chulalongkorn Medical Journal.

[ref-16] Krithong R, Nuinoon M, Pramtong S, Sasuk P, Sarakul O (2020). The modified G6PD deficiency screening test. Accreditation and Quality Assurance.

[ref-17] Kutanan W, Kitpipit T, Phetpeng S, Thanakiatkrai P (2014). Forensic STR loci reveal common genetic ancestry of the Thai-Malay Muslims and Thai Buddhists in the deep Southern region of Thailand. Journal of Human Genetics.

[ref-18] Laosombat V, Sattayasevana B, Janejindamai W, Viprakasit V, Shirakawa T, Nishiyama K, Matsuo M (2005). Molecular heterogeneity of glucose-6-phosphate dehydrogenase (G6PD) variants in the south of Thailand and identification of a novel variant (G6PD Songklanagarind). Blood Cells, Molecules, and Diseases.

[ref-19] Ley B, Bancone G, von Seidlein L, Thriemer K, Richards JS, Domingo GJ, Price RN (2017). Methods for the field evaluation of quantitative G6PD diagnostics: a review. Malaria Journal.

[ref-20] Nuchprayoon I, Sanpavat S, Nuchprayoon S (2002). Glucose-6-phosphate dehydrogenase (G6PD) mutations in Thailand: G6PD Viangchan (871G>A) is the most common deficiency variant in the Thai population. Human Mutation.

[ref-21] Pattaranggoon N, Yimtiang T (2014). The study of prevalence and haematological parameters of G6PD deficiency patient: case report from Trang hospital. Journal of the Medical Association of Thailand = Chotmaihet Thangphaet.

[ref-22] Phompradit P, Kuesap J, Chaijaroenkul W, Rueangweerayut R, Hongkaew Y, Yamnuan R, Na-Bangchang K (2011). Prevalence and distribution of glucose-6-phosphate dehydrogenase (G6PD) variants in Thai and Burmese populations in malaria endemic areas of Thailand. Malaria Journal.

[ref-23] Recht J, Ashley EA, White NJ (2018). Use of primaquine and glucose-6-phosphate dehydrogenase deficiency testing: divergent policies and practices in malaria endemic countries. PLOS Neglected Tropical Diseases.

[ref-24] Suryantoro P (2003). Glucose-6-phosphate dehydrogenase (G6PD) deficiency in Yogyakarta and its surrounding areas. The Southeast Asian Journal of Tropical Medicine and Public Health.

[ref-25] Tanphaichitr VS (1999). Glucose-6-phosphate dehydrogenase deficiency in Thailand; its significance in the newborn. The Southeast Asian Journal of Tropical Medicine and Public Health.

[ref-26] Thielemans L, Gornsawun G, Hanboonkunupakarn B, Paw MK, Porn P, Moo PK, Van Overmeire B, Proux S, Nosten F, McGready R, Carrara VI, Bancone G (2018). Diagnostic performances of the fluorescent spot test for G6PD deficiency in newborns along the Thailand-Myanmar border: a cohort study. Wellcome Open Research.

[ref-27] Tuchinda S, Rucknagel DL, Na-Nakorn S, Wasi P (1968). The Thai variant and the distribution of alleles of 6-phosphogluconate dehydrogenase and the distribution of glucose 6-phosphate dehydrogenase deficiency in Thailand. Biochemical Genetics.

[ref-28] van den Broek L, Heylen E, van den Akker M (2016). Glucose-6-phosphate dehydrogenase deficiency: not exclusively in males. Clinical Case Reports.

[ref-29] Wang J, Matsuoka H, Hirai M, Mu L, Yang L, Luo E (2010). The first case of a class I glucose-6-phosphate dehydrogenase deficiency, G6PD Santiago de Cuba (1339 G > A), in a Chinese population as found in a survey for G6PD deficiency in northeastern and central China. Acta Medica Okayama.

[ref-30] World Health Organization (2015). Guidelines for the treatment of Malaria.

[ref-31] World Health Organization (2022). Technical consultation to review the classification of glucose-6-phosphate dehydrogenase (G6PD).

[ref-32] Yusoff NM, Shirakawa T, Nishiyama K, Ee CK, Isa MN, Matsuo M (2003). G6PD Viangchan and G6PD Mediterranean are the main variants in G6PD deficiency in the Malay population of Malaysia. The Southeast Asian Journal of Tropical Medicine and Public Health.

