# Supplementary figures and images for: Prevalence of G6PD deficiency and G6PD variants amongst the southern Thai population
Source: PeerJ. 2022 Oct 10;10:e14208. doi: 10.7717/peerj.14208 (PMC9559062; doi:10.7717/peerj.14208)

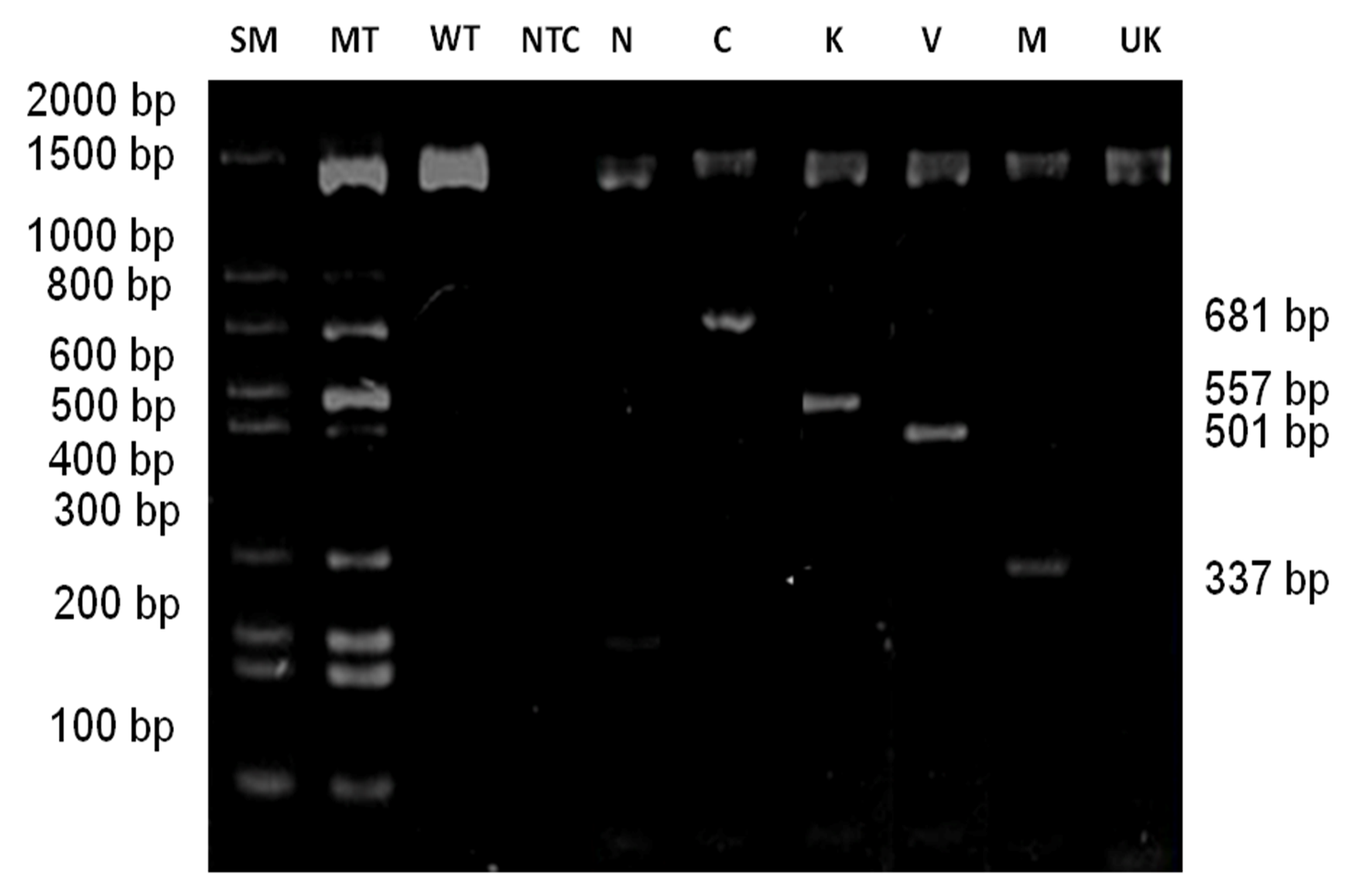

Supplement: Supplemental Information 2 — The pattern of PCR products of G6PD deficiency cases was analyzed by 3% gel electrophoresis. Lane 1, standard marker; lane 2, mutant type control; lane 3, wild type control; lane 4, non-template control; lane 5, G6PD normal; lane 6, G6PD Canton (681 bp); lane 7 G6PD Kaiping (557 bp); lane 8 G6PD Viangchan (501 bp); lane 9 G6PD Mahidol (337 bp) and lane 10 no detectable mutation by PCR used in this study. [file peerj-10-14208-s002.png]

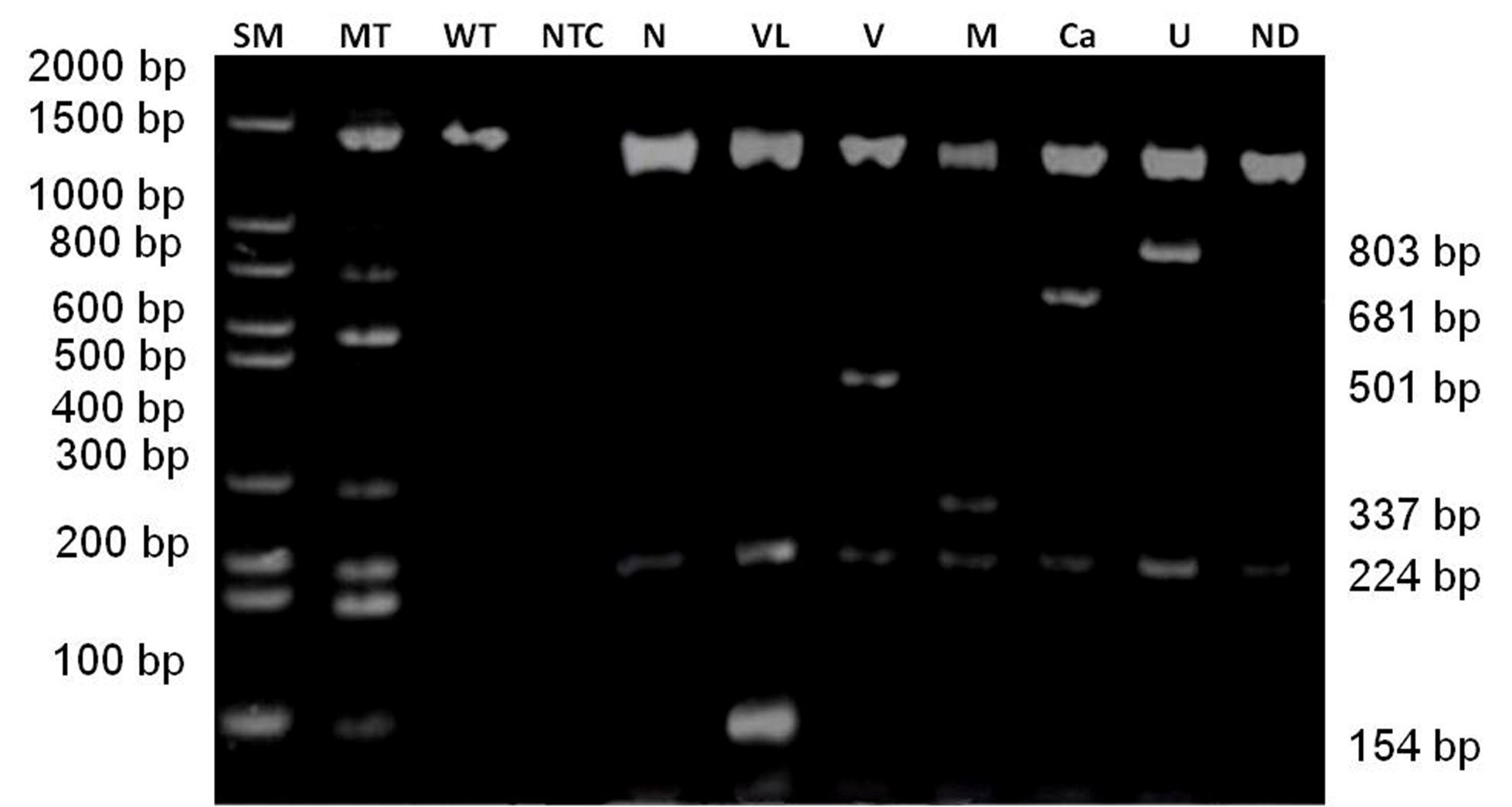

Supplement: Supplemental Information 3 — The pattern of PCR products of G6PD partial deficiency cases analyzed by 3% gel electrophoresis. Lane 1, standard marker; lane 2, mutant type control; lane 3, wild type control; lane 4, non-template control; lane 5, G6PD normal; lane 6, G6PD Vanua Lava (154 bp); lane 7, G6PD Viangchan (501 bp); lane 8, G6PD Mahidol (337 bp); lane 9, Canton (681 bp); lane 10, G6PD Union (803 bp) and lane 11, no detectable mutation by PCR used in this study. [file peerj-10-14208-s003.png]
